# Age and Activity

**Published:** 1901-11-02

**Authors:** 


### The Hospital

A Journal of

The Medical Sciences and Hospital Administration.

Vol. XXXI.—No. 788 ] Edited by Sir Henry Burdett, K.C.B., and Solomon C. Smith, M.D., M.R.C.P. [November 2, 1901.

### Age and Activity.

A Nestor of the profession of surgery, Sir Henry Thompson, who, at the ripe age of 82, retains very considerable bodily and undiminished mental activity, has just given to the world, in an enlarged and amended form, an essay on diet, in relation to age and activity, which he first published some sixteen years ago, and which he has now so far enlarged as to bring down its precepts from the seventh to the eighth decade of human life. There can, we think, be no doubt that an annually increasing number of persons are engaged in qualifying themselves to profit by its teaching; and that the eighth, or even the ninth decade, is now reached more frequently, by both sexes, than was even the seventh a century ago. The lesson to be learnt by anyone who will cast an observing eye down the obituary columns of the newspapers is too plain to require enforcement by the letter of the "Constant Reader," who occasionally informs the editor of the precise number of octogenarians whose deaths were recorded in the issue of the preceding Saturday; and the matter is one concerning which all who run may read. The question of the causes to which this result is chiefly to be attributed is a more complicated one than would generally be supposed, and is, indeed, one of which an assured solution could hardly be attained. The obituary columns of the newspapers contain facts which, in the main, are derived entirely from comparatively wealthy classes of the community; and the tables of the Registrar General, although they include all classes, are nevertheless vitiated, for many purposes, by the necessary inclusion of a large number of feeble folk, of sickly or neglected children, and of drunkards or depraved persons, who swell the total mortality of the nation by figures which are only applicable to a small proportion of its elements. Speaking in very general terms, it may perhaps be said that moderation is the chief source of the increasing longevity of the rich, including in that term all those whose circumstances place them beyond the probability of want; and that prosperity is the chief source of the increasing longevity of the poor, including in that term all those of whom it may be said, in the words of Johnson's epitaph on Levitt, that

> "The modest wants of every day,
> The toil of every day supplied."

In other words, it is important for the continued life of the rich man that he should use discreetly the favours which fortune has bestowed upon him; and it is important for the continued life of the poor man that his earnings should afford him a sufficiency of reasonable comfort in the directions of food, of clothing, of habitation, and of warmth. Abernethy touched the essence of one aspect of the matter when he declared that the best time to eat was, for a rich man, when he could get appetite, and, for a poor man, when he could get food.

Sir Henry Thompson's very edifying lay sermon is preached, of course, to those who fall within the former of Abernethy's two categories, and may almost be regarded as adding, to the words of the earlier master, "provided they don't eat too much." He draws a quite pathetic picture of the mischief which may be wrought by the loving wife, who endeavours to coax her elderly spouse into swallowing this and that compound supposed to be "nutritious," and who, when her well-intended persuasions fail of their effect, feeds him surreptitiously by concealing calf's-foot jelly in his tea. He lays down the perfectly sound principle that what is called "indigestion," as a rule, does not depend upon any fault of the digestive apparatus, but solely upon its being called upon to accomplish work which is beyond its powers; so that the remedy is not to be found in the gastric juices of the pig, or in the ingestion of the various chemically-prepared messes advertised as being digestible or as being nourishing, but in a simple accommodation of the demands made upon the stomach to its capacity for fulfilling them. He would leave the pepsine and the messes to be applied, if at all, by skilled physicians in cases of illness which may possibly require them, and lays down, as of practically universal application, the principle that the elderly person neither requires nor can digest as much food as the young person, and that this principle should govern the arrangements of his life. The total amount of his food should be steadily diminished as age advances, and this total amount should be divided among a larger number of meals than were sufficient for his wants in former days. In other words, not only should the entire daily demand upon the digestion be diminished, but the demand made at any one time should be diminished also. It is commonly asserted, and is by many believed, that the average duration of human life has been increased by dentistry; but Sir Henry inclines to the opinion that the loss or failure of teeth is one of Nature's kindly warnings that the use of them, and by implication the use of foods which require their active exercise, should be diminished in corresponding proportion. The principle which he applies to food, he applies also to all the forms of alcohol; and his contemptuous rejection of the idea that "wine is the milk of old age," reminds us of Sir James Paget's frequent saying that this or that was "as false as a proverb." Sir Henry's little book should win for him the gratitude of all who are approaching those slopes down which he has descended so gracefully; and it has the rare merit that, in the words of a great moralist, the preacher "is the example of his own sermon."

